# Inflammatory myofibroblastic tumor of the small intestine mimicking acute appendicitis: a case report and review of the literature

**DOI:** 10.1186/s13256-016-0880-0

**Published:** 2016-04-19

**Authors:** Alexandra Oeconomopoulou, Yvelise de Verney, Katerina Kanavaki, Kalliopi Stefanaki, Kitty Pavlakis, Christos Salakos

**Affiliations:** 1 Pediatric Department, “IASO” Maternity and Children’s Hospital, 37-39 Kifisias Street, 15123 Marousi, Athens Greece; 2 Pediatric Radiology Department, “IASO” Maternity and Children’s Hospital, 37-39 Kifisias Street, 15123 Marousi, Athens Greece; 3 Department of Pathology, Children’s Hospital “Aghia Sophia”, Thivon & Levadias, 11527 Goudi, Athens Greece; 4 Department of Pathology, “IASO” Maternity and Children’s Hospital, 37-39 Kifisias Street, 15123 Marousi, Athens Greece; 5 Pediatric Surgery Department, “ΑΤΤΙΚΟΝ” University Hospital, National and Kapodistrian University of Athens, 1 Rimini Street, 12462 Haidari, Athens Greece

**Keywords:** Inflammatory myofibroblastic tumor (IMT), Omental-mesenteric myxoid hamartoma (OMMH), Appendicitis, Laparoscopy, Umbilical incision, Childhood

## Abstract

**Background:**

Inflammatory myofibroblastic tumor is a rare tumor of a borderline malignancy. Although it is commonly seen in children, the number of childhood cases in the current literature is limited. The lung is the most commonly affected location. However, cases that have been documented in the mesentery-omentum have mostly been located in the mesentery of the small bowel and not in the antimesenteric edge as in our patient.

**Case presentation:**

A 6-year-old Greek boy was referred to our hospital with acute abdominal pain mimicking appendicitis. Ultrasound and computed tomography revealed a solid mass in the abdomen. The patient underwent laparoscopic resection of the tumor, and histopathology and immunohistochemical analysis favored an omental-mesenteric myxoid hamartoma, which is a variant of an inflammatory myofibroblastic tumor. The patient’s postoperative course was uneventful, and he has been asymptomatic during follow-up.

**Conclusions:**

Inflammatory myofibroblastic tumor of the small intestine is a rare, benign neoplasm in children that should be considered as a possible cause of acute abdomen. A precise diagnosis can be made on the basis of histologic findings. Surgical excision is the treatment of choice.

## Background

Inflammatory myofibroblastic tumors (IMTs) are very rare lesions that most often affect children and young adults [1, 2]. The IMT is a member of a heterogeneous group of soft tissue tumors. IMTs have various names, including inflammatory pseudotumor, plasma cell granuloma, fibrous histiocytoma, solitary mast cell tumor, and fibroxanthoma [3]. The clinical behavior of IMTs is similar to that of tumors of uncertain malignant potential, so IMTs are considered tumors of borderline malignancy [4]. Omental-mesenteric myxoid hamartoma (OMMH) shares many morphologic features with IMT and may represent a variant of IMT, which is still under debate [5].

The lung is the most commonly affected location, but lesions have been reported in a variety of intraabdominal organs [3, 6, 7]. However, small bowel tumors are particularly rare, and even more rarely they are located in the antimesenteric edge of the small bowel [7]. We report this unusual variant of IMT with a rare location in the small intestine, mimicking acute appendicitis. The literature is reviewed, and clinical and pathologic features of this entity are presented, with an emphasis on diagnosis and treatment in children.

## Case presentation

A 6-year-old Greek boy was referred to our hospital with acute abdominal pain, fever, and vomiting that had started 30 h earlier. On presentation, he was hemodynamically stable and well-hydrated. His abdominal examination revealed right lower quadrant pain on palpation and a positive McBurney sign with signs of peritoneal irritation. His hemoglobin was 12.2 g/dl (normal range 12–15 g/dl), his hematocrit was 34.6 % (normal range 36–44 %), his mean corpuscular volume was 72.7 (normal range 77–89), his mean corpuscular hemoglobin was 24.7 (normal range 25–31), his leukocyte count was 15,110/μl (normal range 5000–13,500/μl), his platelet count was 279,000/μl (normal range 200,000–400,000/μl), and his C-reactive protein level was 3.81 mg/dl (normal range <0.51 mg/dl). His electrolyte and coagulation profiles were within normal ranges.

**Fig. 1:**
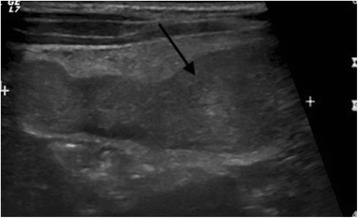
Ultrasound shows a hypoechoic fusiform mass (*arrow*)

**Fig. 2:**
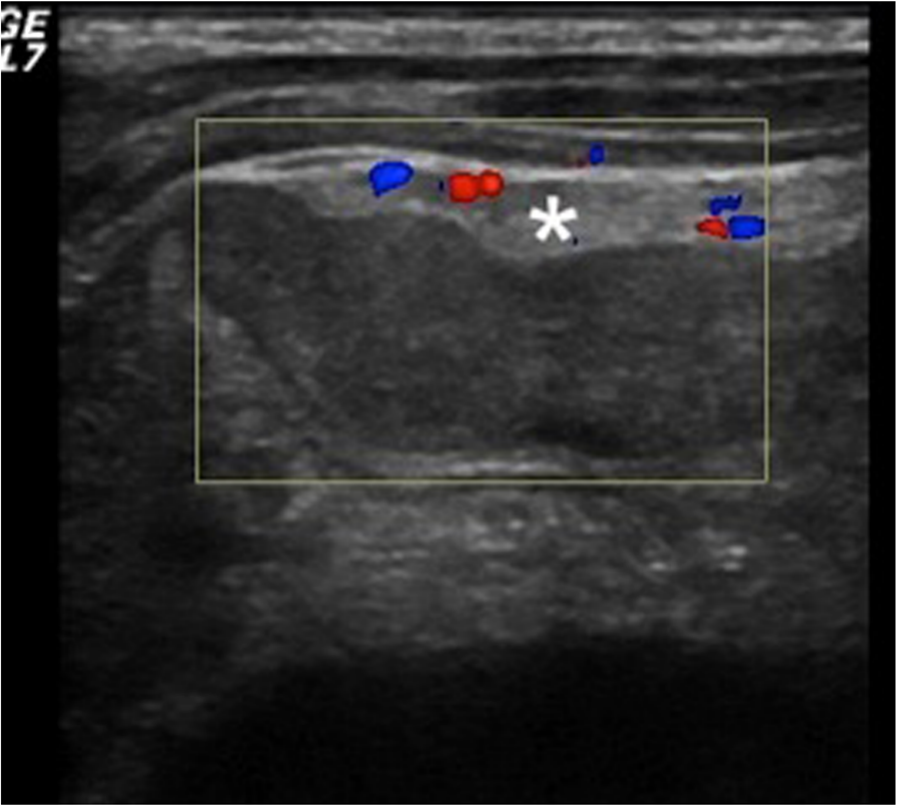
The mass was not vascularized, and surrounding mesenteric fat was hyperechoic and hypervascularized on a color Doppler ultrasound (*asterisk*)

**Fig. 3:**
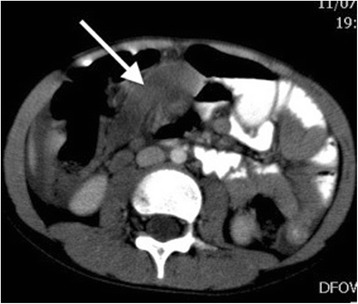
Computed tomographic scan shows a fusiform, nonenhancing solid mass situated between the right hemiabdomen and the midline (*arrow*)

**Fig. 4:**
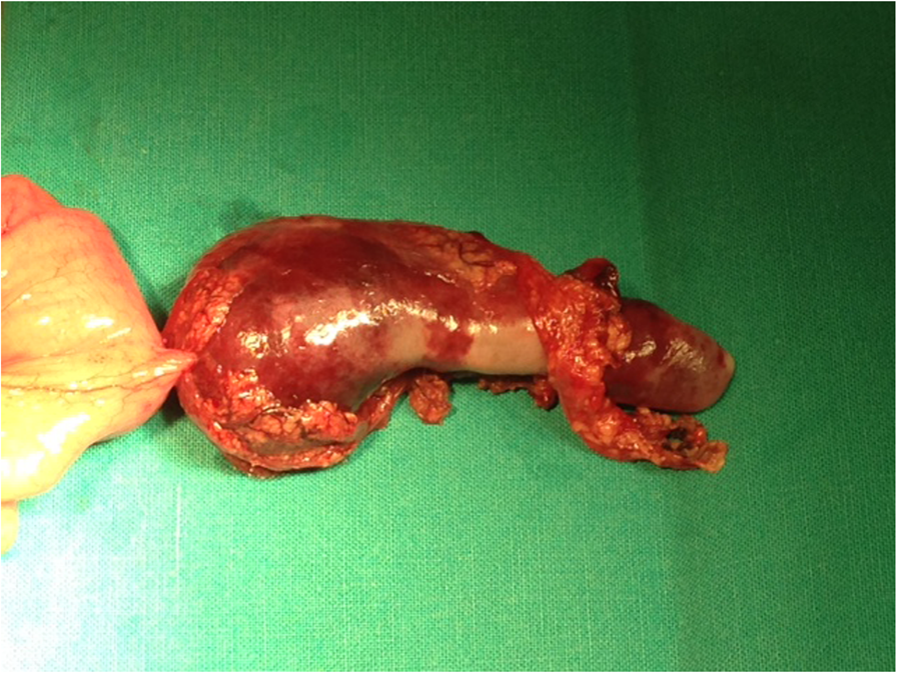
Torsion of the pediculated mass covered by inflammatory omentum in contact with the antimesenteric edge of the small bowel, exteriorized by the umbilical incision

As the boy’s history and physical examination referred to acute appendicitis, ultrasound was performed. Ultrasonography showed a well-defined, hypoechogenic solid mass measuring 6 × 2 cm in the right iliac fossa (Figs. 1 and 2). Other abdominal structures, including the appendix, liver, and kidneys, were normal. Because of the complex nature of the lesion, computed tomography was also performed. A computed tomographic scan showed a solid mass measuring 6 × 2.4 cm in the right abdomen. The mass was in contact at one end with the ascending colon and at the other end with the small bowel (Fig. 3). Imaging findings excluded the presence of acute appendicitis or Meckel’s diverticulum. Emergency laparoscopic exploration was performed using a 10-mm trocar placed in the umbilicus with two accessory trocars in the left and right lower quadrants (5 mm). A pediculated solid mass attached to the antimesenteric edge of the ileum and 8 cm proximal to the ileocecal valve was found. It was covered by inflammatory omentum. It appeared ischemic due to torsion at the level of the pediculated attachment to the ileum. The inflammatory part of the omentum covering the mass was laparoscopically resected free, and the mass with the adjacent ileal loop was exteriorized through a circumbilical incision at the site of the umbilical opening and easily separated from the ileal edge [8]. The ileal wall was normal (Fig. 4).

The histopathologic diagnosis was made by using hematoxylin and eosin-stained slides and immunohistochemistry. The histological examination revealed a circumscribed mesenchymal myofibroblastic lesion with a focal mesothelial lining and a considerable vascular component showing excessive hemorrhage and heterogeneous ischemic necrosis. The lesion was composed of clustered and dispersed fibroblastic spindle cells with eosinophilic cytoplasm and a nucleus with fine chromatin without any considerable nuclear atypia or mitotic activity. Characteristic features were the myxoid configuration of the lesion, the variable hyalinization, and the moderate inflammatory infiltrate composed mainly of plasma cells and lymphocytes.

Immunohistochemistry of the spindle cells showed focal expression of desmin (clone D33), cytokeratins 8 and 18, and pan-keratin AE1/AE3, while there was no detection of smooth muscle actin (SMA), myogenin/Myf-4, CD34, S100, c-kit/CD117, epithelial membrane antigen, β-catenin, Bcl-2 protein, melanosome-associated antigen/HMB45, or anaplastic lymphoma kinase (ALK)-1/p80. Although the described immunophenotype is not entirely diagnostic of a specific entity, it may be encountered in OMMH, which is considered a variant of IMT, though this is a subject of debate.

The patient’s laboratory values improved dramatically after surgery, and he had an uneventful postoperative course. He was discharged from the hospital on the fourth postoperative day. No evidence of recurrence was noted during 2 years of follow-up, and the patient remains under clinical surveillance.

## Discussion

IMT was first described in 1937 as a lung tumor and is more common in children and young adults [4, 6]. IMT is a rare lesion that belongs to the group of soft tissue tumors. The variety of terms used to describe this entity include *inflammatory pseudotumor*, *plasma cell granuloma*, *fibrous histiocytoma*, *solitary mast cell tumor*, and *fibroxanthoma* [3]. OMMH shares many morphologic features with IMT, and it may represent a variant of IMT, which is still under debate [4].

The etiologic factors responsible for the development of IMT are not clearly established [1–3, 6]. IMT may represent an immunologic response to an infectious or noninfectious agent, or it may be a true tumor. *Campylobacter jejuni*, Epstein-Barr virus, and *Escherichia coli* have been associated with IMT. Also, trauma, steroid use, abdominal surgery, and genetic factors have been reported, but the pathogenesis of IMT remains unclear [1].

Presenting symptoms depend on the involved primary site by the tumor. The lung is the most commonly affected site, but extrapulmonary IMT may include the mesentery-omentum, upper respiratory tract, genitourinary tract, retroperitoneum, pelvis, head, neck, spleen, brain, pancreas, liver, and gastrointestinal tract [4, 6]. Documented IMT cases in the mesentery-omentum have been located mostly in the mesentery of the small bowel and not in the antimesenteric edge, as was the case in our patient [7]. Patients with intraabdominal tumors most commonly present with intermittent abdominal pain due to the solid mass and the abdominal distention, as well as with weight loss, malaise, anorexia, and vomiting [3, 7, 9, 10]. Rarely, the presentation may be complicated by an intestinal obstruction, intussusception, or acute abdomen mimicking acute appendicitis, as was the case in our patient [2]. Laboratory abnormalities are present in a minority of patients and include hypochromic microcytic anemia, as in our patient; increased serum immunoglobulins; and elevated thrombocyte counts [2, 6, 7]. Leukocytosis, as in our patient, is a rare hematologic finding that should be included in the list of laboratory abnormalities [1, 2]. After the tumor excision, all of our patient’s symptoms and laboratory values resolved.

Microscopically, a variety of patterns can be seen in IMT. Some lesions consist of a vascularized myxoid stroma with plump mesenchymal cells having vesicular nuclei and prominent nucleoli. Cellular foci alternate with areas of collagenization. Some lesions are characterized by an admixture of lymphocytes and plasma cells. Other lesions are composed of a compact proliferation of spindle-shaped cells arranged in a storiform or fascicular growth pattern. The mitotic rate is low [7]. Immunohistochemistry confirms the final diagnosis when mesenchymal cells are immunoreactive for vimentin, desmin, SMA, and S100 protein and do not express CD34 [6]. Up to 71 % are positive for ALK-1; this immunophenotypic feature is more frequent in younger males and is associated with a high recurrence rate [10, 11].

The differential diagnosis of IMT is difficult even at a microscopic level, and immunohistochemical analysis helps to differentiate IMTs from other tumors, such as gastrointestinal stromal tumors, leiomyosarcomas, and inflammatory malignant fibrous histiocytomas [12].

IMTs generally have a benign course, and recurrence or distant metastasis is rare. The incidence of local recurrence has been reported to be 15–37 % when an IMT is located in the mesentery or the retroperitoneum. Nevertheless, to our knowledge, no precise recommendations exist in the literature on the necessity of any additional therapy [1, 2, 6]. Complete surgical resection remains the treatment of choice for IMT [1, 2, 6, 9, 10].

## Conclusions

IMT is a rare solid tumor that can at times present in a young child as acute abdomen mimicking acute appendicitis. A precise diagnosis should be based on histological findings, and complete surgical resection is necessary. Clinical and laboratory follow-up is mandatory because of an increased risk for local recurrence.

## Consent

Written informed consent was obtained from the patient’s legal guardian for publication of this case report and any accompanying images. A copy of the written consent is available for review by the Editor-in-Chief of this journal.

## Abbreviations

ALK-1: anaplastic lymphoma kinase 1
IMT: inflammatory myofibroblastic tumor
OMMH: omental-mesenteric myxoid hamartoma
SMA: smooth muscle actin
